# Performance Assessment of Two Different Approaches of Measuring Skeletonized Radial Peripapillary Capillary Vessel Density in Glaucoma Patients

**DOI:** 10.3389/fmed.2021.814306

**Published:** 2022-03-16

**Authors:** Yiqin Guo, Yunxiao Sun, Xueyuan Zhang, Ningli Wang

**Affiliations:** ^1^Beijing Tongren Eye Center, Beijing Tongren Hospital, Capital Medical University, Beijing, China; ^2^Beijing Ophthalmology and Visual Science Key Laboratory, Beijing Institute of Ophthalmology, Beijing, China; ^3^Department of Peripheral Vascular Disease, Xiyuan Hospital, China Academy of Chinese Medical Sciences, Beijing, China

**Keywords:** measuring method, OCT angiography, glaucoma, radial peripapillary capillary vessel density, skeletonization

## Abstract

**Objective:**

To compare performance assessment of two methods of measuring radial peripapillary capillary (RPC) vessel density (VD) after skeletonization using MATLAB and Image J in glaucoma clinical setting.

**Methods:**

Seventy-three eyes of 73 glaucoma patients from Beijing Tongren Hospital were included in this prospective study. Original images of RPC were obtained using optical coherence tomography angiography. Two approaches were executed before measuring. Method 1 (M1) required image sharpening, removal of big vessels, and skeletonization. Method 2 (M2) required skeletonization and removal of major vessels. Each method was executed twice. Repeatability and correlations with glaucomatous parameters were assessed. Factors associated with retinal nerve fiber layer thickness (RNFLT) and visual field mean deviation (MD) were analyzed.

**Results:**

Average VD was 13.86 ± 2.73 and 7.50 ± 2.50% measured by M1 and M2. Percentage of total elimination of the major vessels was 36.99 and 100% by M1 and M2, respectively. The intrasession and intersession reproducibility was higher by M2 (ICC = 0.979, ICC = 0.990) than by M1 (ICC = 0.930, ICC = 0.934). VD measured by M2 showed stronger correlations with glaucomatous parameters than by M1. By stepwise multiple linear regression, thinner RNFLT was associated with smaller VD measured by M2 (*B* = 4.643, *P* < 0.001). Worse MD was associated with smaller VD measured by M1 (*B* = 1.079, *P* = 0.015).

**Conclusion:**

The VD measured by M2 showed better reproducibility and higher correlation with glaucomatous structural parameters. Image sharpning helps display of hazy vasculature in glaucoma, which may reflect visual function better. Researchers should carefully choose image processing methods according to their research object.

## Introduction

In the field of research in the vascular mechanism of glaucoma, the system of peripapillary capillaries is of profound interest (1–6). The question remains a mystery whether diminished microvasculature precedes the retinal nerve fiber layer loss or the opposite is true (3, 4). The peripapillary microcirculation in the retina is a paramount indicator for numerous retinal diseases and optic nerve degeneration (7–11), especially for glaucoma (3, 4). Objective and precise assessment of the radial peripapillary capillaries is crucial to the diagnosis of the disease and evaluation of severity and progression (6, 12). Compared to fluorescein angiography (13) and indocyanine green angiography (14), optical coherence tomography (OCTA) is a non-invasive facility that can provide separated imaging of different layers with high resolution, allowing quantification of peripapillary retinal microcirculation (15). AngioVue Software can automatically compute vessel density of peripapillary capillary without major vessels. However, the current available automated software does not provide images of vasculature in specific layer without major vessels, which limits the reprocessing of the images. In addition, bias produced by the artificial moving effects and the variability caused by different vessel calibers could not be avoided using the automated software. With applicable image-analysis software, skeletonization of the vessels can reduce the width of the vessels to a single pixel, eliminating related variability of vessel density caused by different vessel calibers (16). Several studies have measured the VD after skeletonization to study the characteristics of ocular diseases (17–20). However, to the best of our knowledge, there is a lack of study that helps assess the performance of different skeletonization methods and determine which method shows the higher correlations with the clinical findings. With the question of vascular theory of glaucoma, this study aimed to evaluate the performance of two quantitative methods of measuring skeletonized peripapillary VD in glaucoma clinical setting.

At present, one of the most commonly used tools for obtaining skeletonized vasculature images was the function bwmorph in Matlab. This is a useful tool for binarization and skeletonization. However, the selection of major vessels in Matlab will need complex codes that may involve the definition of vessel perimeters or area. The definition is hard to be unified for the variety of morphology of the peripapillary microvasculature in different individuals. Moreover, the random intersection of the vessels constantly causes false selection using the existing algorithm in Matlab. The thresholding technique in Image J is a convenient visualized tool that can help divide an image into two or more classes of pixels according to the intensity of the image. We intended to use this tool to single out the peripapillary major vessels for their higher intensity than the capillaries. In this article, we introduce two methods to obtain skeletonized microvasculature without major vessels using the combination of Image J and Matlab software and compare their performance in the glaucoma clinical setting.

## Methods

This study was approved by the Ethics Committee of Beijing Tongren Hospital and adhered to the tenets of the Helsinki declaration. Subjects were recruited from the glaucoma clinic at Beijing Tongren Hospital from September 2019 to April 2021 (Chinese Clinical Trial Registry No. ChiCTR1800017875). All participants provided written informed consent before entering the study.

All subjects underwent thorough ophthalmic examination including best-corrected visual acuity, intraocular pressure (IOP), refraction test, slit-lamp biomicroscopy, Humphrey visual field test (Humphrey Field Analyzer III, Carl Zeiss Meditec, Inc., SITA fast program, 24-2), fundus examination, OCT and OCTA imaging system (RTVue-XR Avanti, Optovue, Inc., Fremont, CA, United States, software version A 2017,1,0,155). Systolic blood pressure (SBP) and diastolic blood pressure (DBP) were measured at the time of OCTA. The mean arterial pressure (MAP) and ocular perfusion pressure (OPP) were calculated with the following equations: MAP = DBP + 1/3 (SBP-DBP); OPP = 2/3 MAP – IOP at the time of OCTA. Inclusion criteria were: (1) glaucoma patients with age over 18 years old; (2) best-corrected visual acuity better than 0.1; (3) refractive error between −9.0D and +3.0 diopters. Exclusion criteria included: (1) quality scores <4; (2) severe opacity in the optical media including serious cataract or vitreous opacity that blocked the imaging of peripapillary vessels. (3) other retinal diseases other than glaucoma. Glaucoma was diagnosed if glaucomatous optic nerve damage was diagnosed by OCT accompanied by corresponding visual field defects determined by two eligible visual field tests (fixation loss <30%, false-positive and false-negative errors lower than 20%).

### OCTA Image Acquirement

The OCTA test was performed on randomly selected glaucomatous eyes for all subjects *via* undilated pupils. The HD Angio Disc protocol was used to acquire peripapillary RPC vasculature images in a 4.5 × 4.5 mm square centered on the optic disc with high resolution. This protocol allows 400 B-scans equally spaced on the X and Y axis. Each B-scan has 400 A-scans. Images of RPCs were stratified from the internal limiting membrane (ILM) to the posterior border of the retinal nerve fiber layer (RNFL). The margins of the optic disc were manually adjusted according to the en face images of the optic nerve head.

The average peripapillary RNFLT, cup/disc area ratio, cup/disc vertical ratio, rim and cup volume, disc elevation, and cup depth were automatically calculated by OCTA software based on 3D OCTA intensity images of the discs, derived from the RNFL slab.

Images of low quality were excluded if: (1) quality scores <4; (2) opacity or floaters caused local blockage of the imaging; (3) Severe artifacts influenced the patterns of the vessels. (4) RNFLT segmentation errors.

### Two Methods of Measuring Skeletonized Vessel Density Using MATLAB and Image J

Method 1 (M1) used Image J to sharpen the original images first in order to enhance the outline of the faint RPCs in glaucoma patients. The sharpened images were then binarized after being adjusted to 8-bit mode and saved as the first images (Image A). The next step required operation on the original images using the “Smooth” function twice or three times to dim the vessels. The major retinal vessels were selected by using the “Threshold” function after being transformed to 8-bit mode. Otsu Method was used and the threshold was adjusted to select only major vessels. Black foreground and white background were made and the achieved pictures were saved as the second images (Image B). Then the MATLAB was used to acquire skeletonized RPC images without major retinal vessels using Image A to subtract Image B.

Method 2 (M2) used Image J to skeletonize the images first after transformation to 8-bit and binarization and saved the images as “Image A.” The next step was the same as M1 to acquire “Image B.” Then the MATLAB was utilized to acquire the final image using Image A to subtract Image B.

VD was defined as the percentage of the area occupied by RPCs without major retinal vessels. VD was measured using Image J after establishing a 750 μm annulus extending from the disc margin.

The acquirement of skeletonized images and the measurement of VD was carried out by the same researcher (G. YQ) twice for analysis of intra-grader repeatability and by another researcher (Z. XY) for analysis of inter-grader repeatability.

### Statistical Analysis

Data analysis was performed using SPSS (version 25, SPSS Inc., Chicago, USA). The repeatability of the measurement was assessed using the intraclass correlation coefficient. The correlations between glaucomatous parameters and VD measured by different methods were evaluated using the Pearson test for normally distributed data and the Spearman test for abnormally distributed statistics. Factors associated with visual field MD were selected by stepwise multiple linear regression. Independent variables were age, sex, intraocular pressure (IOP), ocular perfusion pressure (OPP), axial length (AL), central cornea thickness (CCT), RNFLT, cup/disc vertical ratio, cup/disc area ratio, rim volume, spherical equivalent (SE), systolic blood pressure (SBP), diastolic blood pressure (DBP), mean arterial pressure (MAP), VD measured by M1 and VD measured by M2. Factors associated with RNFLT were selected by stepwise multiple linear regression. Independent variables were age, sex, IOP, OPP, AL, CCT, visual field MD, cup/disc vertical ratio, cup/disc area ratio, rim volume, SE, SBP, DBP, MAP, VD measured by M1, and VD measured by M2. Data are presented as mean ± standard deviation. Statistical significance was set at *P* < 0.05.

## Results

In total, 80 eyes underwent OCTA examinations, 7 of which were excluded due to poor quality OCTA images. Table 1 shows the demographic characteristics of the subjects. 42 males and 31 females were included. The mean age was 47.37 ± 13.71 years old, ranging from 20 to 81. The average AL were 25.15 ± 1.58 mm, with a mean spherical equivalent of −3.75±2.97 D. IOP ranged from 11.67 to 31.5 mmHg, with an average value of 17.37 ± 4.56 mmHg. The mean CCT was 524.25 ± 31.57 μm. The average RNFLT was 76.84 ± 17.64 μm and the mean visual field MD was −9.38 ± 7.48 dB.

**Table 1:** Demographic characteristics.

**Characteristics**	**Subjects (*n* = 73,** **eyes = 73)**
Gender	
Male, no.	42
Female, no.	31
Age, years	
Mean ± SD, yrs	47.37 ± 13.71
Range, yrs	20–81
Ocular characteristics	
AL, mm	25.15 ± 1.58
SE, diopters	−3.75 ± 2.97
IOP, mmHg	17.37 ± 4.56
OPP, mmHg	43.67 ± 8.70
CCT, μm	524.25 ± 31.57
Cup/disc area ratio	0.45 ± 0.18
Cup/disc vertical ratio	0.68 ± 0.19
Cup volume, mm^3^	0.21 ± 0.16
Rim volume, mm^3^	0.19 ± 0.22
Disc elevation, μm	−9.99 ± 143.98
Average peripapillary RNFLT, μm	76.84 ± 17.64
Visual field MD, dB	−9.38 ± 7.48
Systemic characteristics	
SBP, mmHg	121.36 ± 16.08
DBP, mmHg	76.62 ± 10.52
MAP, mmHg	91.53 ± 11.57

*SD, Standard Deviation; AL, Axial length; SE, Spherical equivalent; IOP, Intraocular pressure; OPP, Ocular perfusion pressure; CCT, Central cornea thickness; RNFLT, Retinal nerve fiber layer thickness; MD, Mean deviation; SBP, Systolic blood pressure; DBP, Diastolic blood pressure; MAP, Mean arterial pressure*.

Table 2 illustrates the repeatability of the two different quantitative methods. Both of the two methods show high reproducibility. M2 showed higher intrasession and intersession repeatability for the measurement of average peripapillary VD (ICC = 0.979, ICC = 0.990) than M1 (ICC = 0.930, ICC = 0.934). Lower value was obtained by M2 (average of 7.50 ± 2.50%) than by M1 (average of 13.86 ± 2.73%). Comparing the skeletonized images by M1 and M2, there were outlines of major vessels remaining after processing by M1 (46 cases, 63.01%), whereas the clearance of big vessels by M2 (0 cases, 0%) appeared perfect.

**Table 2:** Intraclass correlation coefficients of two quantitative methods.

	**Average RPC** **vessel density**	**Intrasession**		**Intersession**	
		**repeatability**		**repeatability**	
	**Mean ±SD, %**	**ICC**	***P***	**ICC**	***P***
M1	13.86 ± 2.73	0.930	**<0.001**	0.934	**<0.001**
M2	7.50 ± 2.50	0.979	**<0.001**	0.990	**<0.001**

*RPC, Radial peripapillary capillary; M1, Method 1; M2, Method 2; SD, Standard deviation; ICC, Interclass Correlation Coefficient.*

*Boldface P values indicate statistically significant difference at p < 0.05*.

Table 3 showed correlations between the glaucomatous parameters and the results of VD by two different methods. VD measured by M2 showed significantly negative correlation with cup volume (*r* = −0.233, *p* = 0.047), whereas VD by M1 did not (*r* = −0.17, *p* = 0.15). Higher correlations were found between VD quantified by M2 and rim volume (*r* = 0.622, *p* < 0.001), cup/disc area ratio (*r* = −0.438, *p* < 0.001), cup/disc vertical ratio (*r* = −0.484, *p* < 0.001) and disc elevation (*r* = 0.394, *p* = 0.001), compared to the result of M1 (*r* = 0.616, *p* < 0.001; *r* = −0.394, *p* = 0.001; *r* = −0.465, *p* < 0.001; *r* = 0.245, *p* = 0.036, respectively).

**Table 3:** Correlations between radial peripapillary capillary vessel density measured by different methods and glaucomatous parameters.

**Parameters**	**RPC vessel**		**RPC vessel**	
	**density measured**		**density measured**	
	**by M1**		**by M2**	
	***r***	***P***	***r***	***P***
IOP	−0.193	0.105^*^	−0.2	0.092^*^
Cup volume	−0.17	0.15^†^	−0.233	**0.047** ^*^
Rim volume	0.616	**<0.001** ^*^	0.622	**<0.001** ^*^
Cup/disc area ratio	−0.394	**0.001** ^†^	−0.438	**<0.001** ^*^
Cup/disc vertical ratio	−0.465	**<0.001** ^*^	−0.484	**<0.001** ^*^
Disc elevation	0.245	**0.036** ^†^	0.394	**0.001** ^*^
Cup depth	−0.048	0.686^†^	−0.055	0.643^*^

*RPC, radial peripapillary capillary; M1, Method 1; M2, Method 2; IOP, Intraocular pressure.*

* *The P-value was determined by Pearson correlation test.*

† *The P-value was determined by Spearman correlation test.*

*Boldface P values indicate statistically significant difference at p < 0.05*.

Tables 4, 5 illustrate factors associated with RNFLT and visual field MD according to stepwise multiple linear regression. Smaller average VD measured by M2 (*B* = 4.643, *P* < 0.001), worse visual field MD (*B* = 0.523, *P* = 0.008), smaller rim volume (*B* = 16.319, *P* = 0.002), and smaller SE (*B* = 1.188, *P* = 0.003) were significantly associated with thinner RNFLT. Worse visual field MD was associated with smaller peripapillary VD measured by M1 (*B* = 1.079, *P* = 0.015), thinner RNFLT (*B* = 0.135, *P* = 0.043), and female gender (*B* = −3.485, *P* = 0.017).

**Table 4:** Factors associated with retinal nerve fiber layer thickness.

**Demographic characteristics**	***B***	**95% confidence Interval**	***P*-value**	**Beta**
Visual field MD	0.523	0.141, 0.905	**0.008**	0.221
Vessel density by M2	4.643	3.464, 5.823	**<0.001**	0.641
SE	1.188	0.413, 1.964	**0.003**	0.197
Rim volume	16.319	6.121, 26.516	**0.002**	0.207

*MD, Mean deviation; M2, Method 2; SE, Spherical equivalent.*

*Boldface P values indicate statistically significant difference at p < 0.05*.

**Table 5:** Factors associated with visual field mean deviation.

**Demographic characteristics**	***B***	**95% confidence Interval**	***P*-value**	**Beta**
Sex	−3.485	−6.323, −0.647	**0.017**	−0.226
RNFLT	0.135	0.004, 0.266	**0.043**	0.32
Vessel density by M1	1.079	0.215, 1.942	**0.015**	0.388

*RNFLT, Retinal nerve fiber layer thickness; M1, Method 1.*

*Boldface P values indicate statistically significant difference at p < 0.05*.

## Discussion

**Figure 1:**
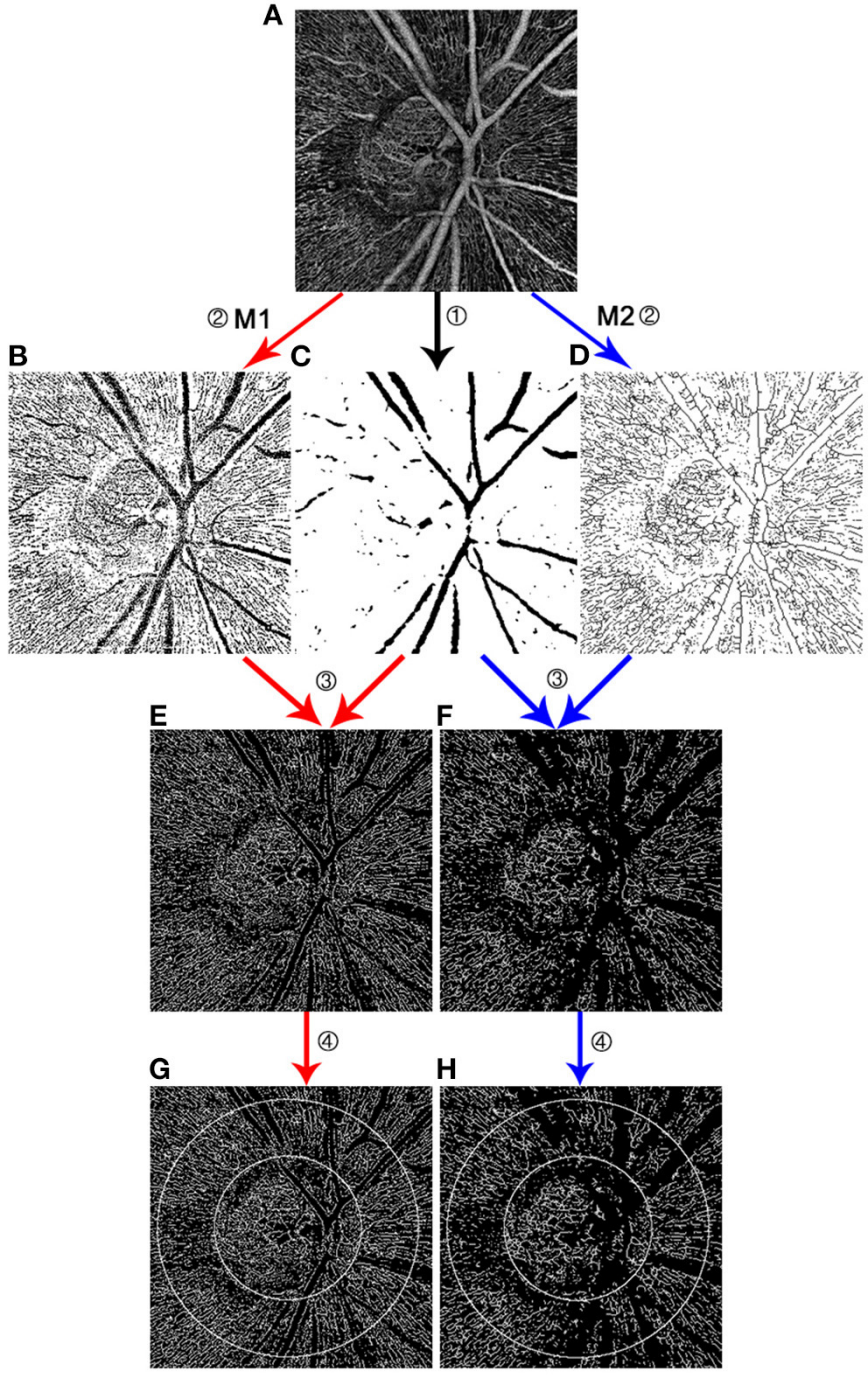
Comparison of two methods measuring radial peripapillary capillary vessel density. Both M1 and M2 required to process on original images output by OCTA **(A)**. ① Through Image J, the extraction of major vessels was obtained using threshold tool **(C)**. ② M1 binarized images after sharpening **(B)**, while M2 binarized and skeletonized the images at this process **(D)**. ③ Using the same MATLAB code, the skeletonized images with the removal of big vessels were obtained. Image **(E)** was the result of image **(B)** minus image **(C)**. Image **(F)** was the result of image **(D)** minus image **(C)**. Outlines of the big vessels were better eliminated in the image **(F)** by M2 than in the image **(E)** by M1. ④ A 750 μm annulus was established extending from the disc margin **(G,H)**.

**Figure 2:**
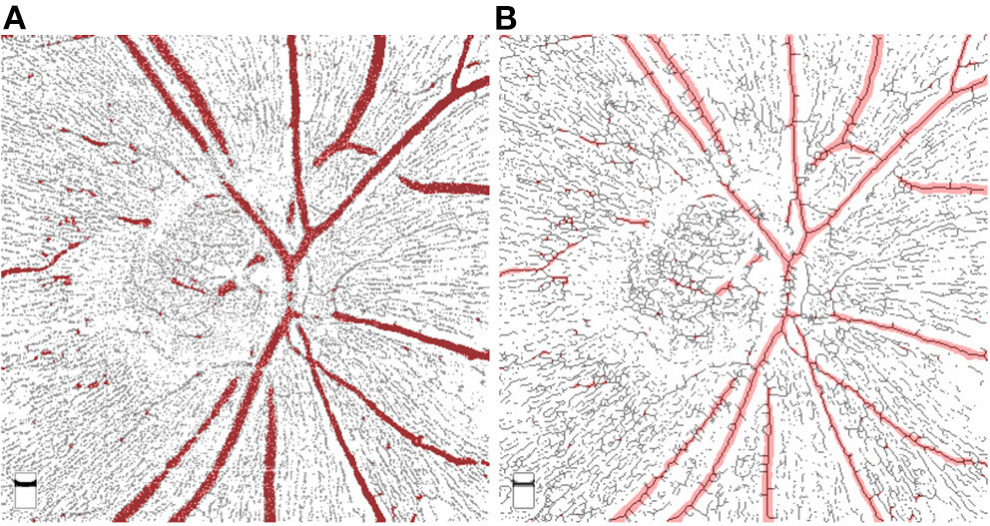
Comparison of superimposed images by Method 1 and Method 2. The selected major vessels (shown in red color) were superimposed on the images processed by M1 **(A)** and M2 **(B)**. As can be seen in **(A)**, the selected big vessels were slightly thinner than the ones obtained by M1, which resulted in the remain of big vessel margins after substraction. However, by using M2, big vessels were skeletonized and well-covered by the selected major vessels.

Skeletonization is a process that makes the width of the vessels narrowed down to a single pixel, therefore the measurement of VD can avoid the influence produced by different vessels calibers (16). Based on the concept of skeletonization, we demonstrated the performance assessment of two different approaches for quantifying RPC vessel density using Image J and MATLAB. In terms of repeatability, the result of M2 showed higher intrasession and intersession reproducibility than M1. The reason for this outcome might mainly result from the variation of selecting major vessels. In the process of selection, major vessels can be chosen by adjusting the maximum and minimum values of the threshold according to the intensity. Usually, the big vessels are dominant with higher intensity compared to peripheral capillaries. However, sometimes part of the capillaries also presents with relatively high intensity, causing infiltration of selection. To reduce this effect, up-regulate the minimum threshold value was needed. Consequently, the margins of big vessels were omitted due to dimness. Thus, the major vessels selected by “threshold” were usually thinner than they should be (Figure 1C). This resulted in the relatively poor performance of clearing out major vessels for M1. By using M1, the intra- and inter-grader repeatability was influenced when major vessels were selected with different calibers at each time and by different researcher. Comparatively, M2 showed better performance in eliminating the margins as can be seen in Figure 2. This was because that M2 required skeletonization first. After skeletonizing, all vessels were processed into lines with a width of a single pixel. Using Skeletonized images to subtract major vessels selected by “threshold” helped obtain images with nearly complete clearance of big vessels.

Comparing the performance of M1 and M2 in the clinical setting, VD measured by M2 showed slightly higher correlations with some glaucomatous parameters, including rim volume, cup/disc vertical ratio, cup/disc area ratio, and disc elevation. In addition, stepwise multiple linear regression screened out VD measured by M2 instead of M1 as one of the associated factors for RNFLT. These results may be related to the better performance of eliminating the major vessels using M2. The retinal structure has a specific association with microvasculature no matter in glaucoma or in healthy subjects. In this term, the influence by variation of big vessels may need attention. However, factors associated with visual field MD included VD measured by M1 rather than by M2. This might because of the sharpening of the images by M1. Due to the massive loss of peripapillary microvasculature in glaucoma, the imaging of capillaries especially in the site of RNFL defect appeared hazy and faint. Some remaining capillaries with low signal may be recognized as no vessels during the process of binarization. Through the process of sharpening, many details were clearly displayed, but so did the artifacts. Thus, a lower occurrence rate of artifact is essential for choosing this method to measure VD. As can be seen in Figure 1 picture E and F, hazy capillaries were sharpened and displayed better by M1. It is acknowledged that better visual function is closely related to the sufficient retinal blood supply. The clear and complete display of blood supply is the strength of M1.

To the best of our knowledge, although skeletonization has already been used in some vascular research (18–20), our study is the first to compare the performance of different approaches for skeletonization. Through the comparison of the two approaches in the clinical setting, an alert has been aroused that different image processing procedures might influence the analysis. Attention should be paid for researchers that proper approach should be carefully chosen according to the object of the research.

There are several limitations of this study. Only glaucoma patients were included in the study. The performance of different processes for skeletonization may differ when it comes to healthy subjects or eyes with other retinal diseases. Relatively small samples may cause boundedness. Besides, both of the approaches have strengths and weaknesses. More complete and convenient methods for ophthalmologists are needed to overcome the limitation of application in batch processing for large sample studies.

In conclusion, the VD measured by M2 showed better repeatability and higher correlation with glaucomatous structural parameters. Image sharpening before skeletonization may better display the hazy vasculature of RPC in glaucoma patients, which may help better reflect visual function. Researchers should carefully choose image processing methods according to their own research object.

## Data Availability Statement

The original contributions presented in the study are included in the article/supplementary material, further inquiries can be directed to the corresponding author.

## Ethics Statement

The studies involving human participants were reviewed and approved by the Ethics Committee of Beijing Tongren Hospital. The patients/participants provided their written informed consent to participate in this study.

## Author Contributions

YG and NW designed the study. YG and YS conducted the examinations and measurements. XZ participated in the measurement and analysis of the data. YG wrote the initial manuscript. NW supervised the study. All authors contributed to the article and approved the submitted version.

## Funding

The authors thank Beijing Tongren Hospital and Beijing Institute of Ophthalmology for the support for this study. The study was funded by Beijing Municipal of Health Reform and Development Project, #2019-4 (Beijing, China).

## Conflict of Interest

The authors declare that the research was conducted in the absence of any commercial or financial relationships that could be construed as a potential conflict of interest.

## Publisher's Note

All claims expressed in this article are solely those of the authors and do not necessarily represent those of their affiliated organizations, or those of the publisher, the editors and the reviewers. Any product that may be evaluated in this article, or claim that may be made by its manufacturer, is not guaranteed or endorsed by the publisher.
